# Role of the protease-activated receptor 2 in multi-walled carbon nanotube-induced macrophage polarization *ex vivo* and airway fibrosis in murine allergic lung disease *in vivo*

**DOI:** 10.3389/ftox.2026.1751684

**Published:** 2026-02-18

**Authors:** Logan J. Tisch, Ryan D. Bartone, Silvio Antoniak, James C. Bonner

**Affiliations:** 1 Toxicology Program, Department of Biological Sciences, North Carolina State University, Raleigh, NC, United States; 2 UNC Blood Research Center, Department of Pathology and Laboratory Medicine, School of Medicine, University of North Carolina at Chapel Hill, Chapel Hill, NC, United States

**Keywords:** allergens, asthma, house dust mite, macrophages, multi-walled carbon nanotubes, protease-activated receptor 2

## Abstract

**Background:**

Our previous work demonstrated that co-exposure to multi-walled carbon nanotubes (MWCNTs) exacerbates allergic lung disease induced by house dust mite (HDM) extract, and that mice deficient in protease-activated receptor 2 (PAR2) show less airway fibrosis following co-exposure. In this study, we examined the role of PAR2 in mediating macrophage polarization in the presence of MWCNTs under Th2-like conditions and the subsequent effects on fibroblast activation *in vitro* and collagen deposition *in vivo*.

**Methods:**

Bone marrow-derived macrophages (BMDMs), murine *ex vivo* alveolar macrophages (mexAMs), and mouse lung fibroblasts (MLFs) were isolated from wild-type (WT) and PAR2 knockout (KO) mice. Macrophages were pretreated with IL-4/IL-13 before exposure to MWCNTs, and polarization markers were analyzed through flow cytometry and Western blot analysis. Conditioned media from treated macrophages were applied to MLFs to assess fibroblast activation. *In vivo*, WT and myeloid-specific PAR2 KO mice were co-exposed to MWCNTs and HDM extract over 21 days, followed by analysis of bronchoalveolar lavage fluid (BALF) and lung tissue for markers of mucous cell metaplasia and airway fibrosis.

**Results:**

MWCNTs exacerbated IL-4/IL-13-induced M2 polarization, increasing Arg-1 and phosphorylated STAT6 levels in both BMDMs and mexAMs. This enhancement was attenuated in PAR2-deficient macrophages. Conditioned media from M2-polarized WT macrophages induced significantly higher expression of profibrotic genes, including *Col1a1* and *Col1a2*, in MLFs compared to conditioned media from PAR2 KO macrophages. *In vivo*, myeloid-specific PAR2 deletion significantly decreased lung collagen deposition and mucus hypersecretion induced by MWCNT and HDM extract co-exposure.

**Conclusion:**

MWCNT exposure exacerbates Th2-driven M2 macrophage polarization in a PAR2-dependent manner, leading to increased fibroblast activation and collagen deposition. Myeloid PAR2 is a critical driver of fibrotic remodeling in allergic lung disease, representing a potential therapeutic target for mitigating fibrosis in environmentally exacerbated asthma.

## Graphical Abstract

**Figure:**
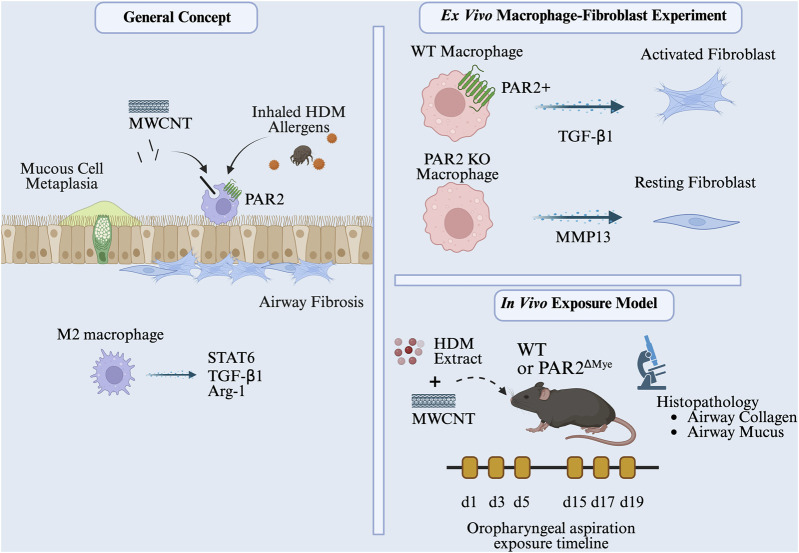

## Introduction

The prevalence of chronic respiratory diseases such as asthma, chronic obstructive pulmonary disease (COPD), and pulmonary fibrosis has significantly increased over the past few decades (Darrow et al., 2014). Epidemiological studies have identified airborne particles as a key environmental factor that worsens the severity of respiratory diseases (Dorion et al., 2019; Kanchongkittiphon et al., 2015). Of particular concern are inhaled particles smaller than 0.1 μm in diameter (i.e., nanoparticles), which can penetrate deep into the lungs and interact with resident immune cells (Duke and Bonner, 2018). Among these particles, engineered nanomaterials (ENMs) such as multi-walled carbon nanotubes (MWCNTs) present a unique toxicological challenge due to their physicochemical properties and increasing industrial use (Cha et al., 2013). Human exposure to MWCNTs occurs predominantly in occupational and industrial settings, particularly during manufacturing and downstream processing. Occupational exposure studies have reported airborne MWCNT concentrations as high as 1,050 μg/m^3^ during secondary manufacturing, while background concentrations in manufacturing facilities typically range from 0.002 to 24.9 μg/m^3^ (Lee et al., 2010; Dahm et al., 2012). Due to their nanoscale size and aerodynamic properties, MWCNTs readily become airborne and are inhaled, making the lung the primary target organ for toxicity.

MWCNTs elicit robust pulmonary immune responses, including eosinophilic inflammation, mucus hypersecretion, and increased collagen deposition when inhaled or co-administered with allergens (Shvedova et al., 2005; Ryman-Rasmussen et al., 2009). Our group has shown that co-exposure to MWCNTs and house dust mite (HDM) extract exacerbates allergic lung disease by amplifying eosinophilic inflammation, mucus production, and collagen deposition (Lee et al., 2023; Tisch et al., 2025a; Tisch et al., 2025b). These outcomes are partly due to the persistence of MWCNTs in lung tissue and their ability to activate profibrotic signaling pathways through the production of cytokines such as transforming growth factor beta 1 (TGF-β1) and platelet-derived growth factor (PDGF) (Duke and Bonner, 2018). Despite these findings, the cellular targets of MWCNTs and the mechanisms by which they worsen allergic lung disease remain unclear. Of particular interest is the protease-activated receptor 2 (PAR2), a G protein–coupled receptor expressed on multiple lung cell types, including epithelial cells, fibroblasts, and innate immune cells such as macrophages. PAR2 has emerged as an important mediator of pulmonary inflammation and fibrosis (Janeway and Medzhitov, 2002; Cocks and Moffatt, 2001; Rayees et al., 2020). PAR2 is activated by endogenous proteases (e.g., tryptase, trypsin, elastase) as well as exogenous allergen proteases such as those found in HDM extract (e.g., Der p1, Der p3) (Sun et al., 2001; Asokananthan et al., 2002). Previous studies show that PAR2-deficient mice are protected from bleomycin-induced pulmonary fibrosis, whereas PAR2 activation promotes fibroblast migration and extracellular matrix production (Lin et al., 2015). Our group has demonstrated that co-exposure to HDM extract and MWCNTs exacerbates allergic lung disease in wild-type (WT) mice compared to PAR2-deficient mice, with WT mice displaying significantly more collagen deposition around the airways (Lee et al., 2023; Tisch et al., 2025a). However, it remains unclear how PAR2 contributes to the cellular and molecular mechanisms underlying inflammation and fibrosis during allergic lung disease, particularly in the context of MWCNT exposure, where the key responding cell types are not fully defined.

Macrophages are of particular interest for mediating these effects because they are among the first immune cells to encounter inhaled particles, and PAR2 signaling within macrophages may regulate the balance between inflammatory and fibrotic responses (You et al., 2020). In response to environmental cues, macrophages can polarize into a classically activated M1 proinflammatory phenotype or an alternatively activated M2 anti-inflammatory and tissue-remodeling phenotype (Rath et al., 2014; Wynn and Barron, 2010). While T cell-derived cytokines such as IL-4 and IL-13 drive polarization during immune activation, macrophages can also polarize independently of T cells in response to signals from the surrounding tissue microenvironment (Goda et al., 2020). This ability to respond directly to environmental and inflammatory stimuli enables macrophages to modulate the balance between Th1 and Th2 immune responses, thereby influencing disease outcomes in the lungs. Recent studies have highlighted the crucial role of macrophages at all stages of lung injury and repair, including their ability to both promote and resolve pulmonary fibrosis (Liu et al., 2019; Lambrecht and Hammad, 2015; Ma et al., 2012). M2 macrophages drive fibrotic remodeling via expression of profibrotic mediators and enzymes such as arginase-1 (Arg-1) (Krane, 2008). Arg-1 is a hallmark of M2 polarization and plays a central role in nitrogen metabolism and collagen biosynthesis by converting L-arginine into L-ornithine, a precursor of proline and hydroxyproline, both essential amino acids for collagen production (Krane, 2008; Morris, 2007). In fibrotic lungs, Arg-1 expression supports tissue remodeling and scar formation by promoting extracellular matrix deposition and fibroblast proliferation, particularly through downstream mediators such as TGF-β1 and PDGF (Escher et al., 2004; Sheppard, 2006). Mounting evidence indicates that MWCNTs can directly modulate macrophage activation and polarization. Macrophages are capable of phagocytosing MWCNTs, and their phenotypic responses depend on the physicochemical properties of the particle and the surrounding immune environment (Boyles et al., 2015; Dong and Ma, 2018a; You et al., 2020). These interactions may skew macrophages toward a profibrotic M2 phenotype, thereby sustaining inflammation and matrix deposition. Thus, MWCNT-induced M2 polarization may be a critical mechanism by which these nanomaterials exacerbate allergic lung disease.

In this study, we investigated the role of PAR2 in modulating macrophage responses to MWCNTs under allergic (Th2-like) conditions and the downstream consequences for fibroblast activation and lung fibrosis. Using *in vitro* models, we treated bone marrow–derived macrophages (BMDMs) and murine *ex vivo* alveolar macrophages (mexAMs) isolated from WT and PAR2 knockout (KO) mice with IL-4 and IL-13 to simulate a Th2 microenvironment, followed by exposure to MWCNTs. Macrophage polarization was assessed by using a combination of Western blot analysis and flow cytometry. To evaluate functional outcomes, primary mouse lung fibroblasts (MLFs) were exposed to macrophage-conditioned media and analyzed for the expression of profibrotic genes, *Arg-1*, *Col1a1*, and *Col1a2*. To extend these findings *in vivo*, we utilized a myeloid-specific PAR2 KO (PAR2^ΔMye^) mouse model to determine the role of PAR2-expressing innate immune cells, primarily macrophages, in allergen- and MWCNT-induced lung disease. We hypothesized that PAR2 promotes MWCNT-induced M2 macrophage polarization and fibroblast activation in a Th2 microenvironment, thereby driving the exacerbation of allergic lung disease.

## Methods

### Multi-walled carbon nanotubes (MWCNTs) and house dust mite (HDM) extract

**FIGURE 1:**
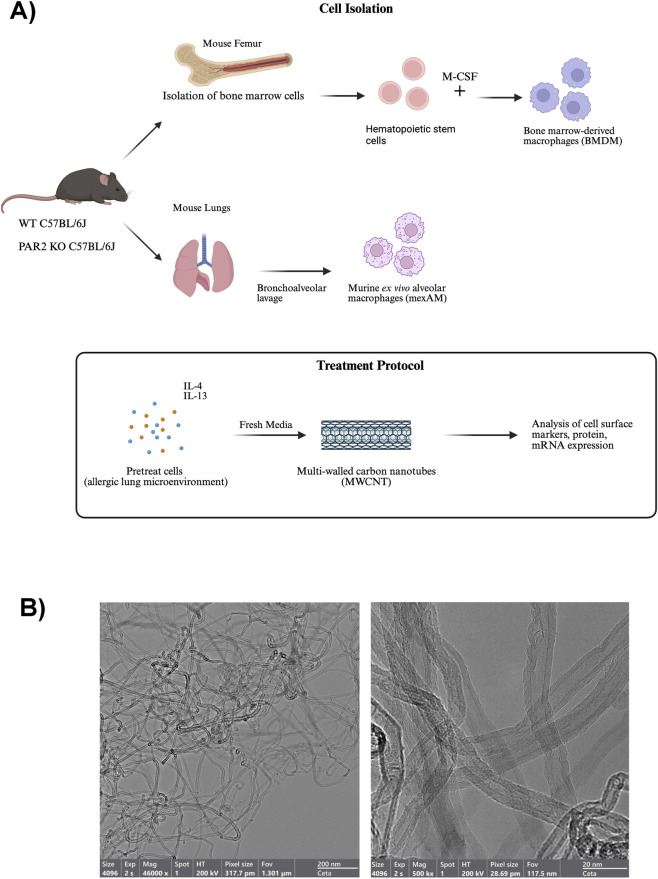
**(A)** Schematic illustration of isolation procedures for bone marrow-derived macrophages (BMDM) and murine *ex vivo* alveolar macrophages (mexAM) from wild-type and PAR2 knockout mice (Created with Biorender.com). **(B)** Representative TEM images of NC7000 MWCNTs.

MWCNTs (NC7000) were purchased from Nanocyl, Inc. and suspended in DPBS. The physicochemical characteristics of NC7000 are listed in Supplementary Image 1 and have been previously published (Taylor-Just et al., 2020). Representative TEM images of NC7000 MWCNTs are shown in Figure 1B. The stock suspension was sonicated in a cup horn sonicator (Q500, Qsonica) for 10 min at 60 amps, then diluted with DPBS to reach the required working concentration for dosing. HDM extract from *Dermatophagoides pteronyssinus* was purchased from Greer Laboratories Inc. and dissolved in DPBS. The stock solution of HDM extract [item #XPB91D3A2.5; lot #390991] contained 1610 endotoxin units (EU).

### Bone marrow-derived macrophage (BMDM) isolation

WT C57BL/6J mice and PAR2 KO C57BL/6J mice were obtained from The Jackson Laboratory (Lee et al., 2023). Mice were sacrificed between 8 and 12 weeks of age, and hind legs were removed for bone marrow isolation. Bone marrow was flushed from the femur and tibia and filtered through a 70-μm cell strainer. The resulting cell mixture was cultured in T-75 tissue culture flasks supplemented with BMDM-differentiation media (DMEM, 10% fetal bovine serum, 1% penicillin/streptomycin, 20 ng/mL M-CSF, PeproTech). Flow cytometry confirmed a phenotypically homogeneous macrophage population and analyses were restricted to cells within established macrophage gates.

### Alveolar macrophage (mexAM) isolation

MexAMs were obtained from WT and PAR2 KO C57BL/6J mice (8–12 weeks of age) from The Jackson Laboratory by bronchoalveolar lavage and cultured as previously described (Kendall et al., 2023). Briefly, the lungs were lavaged with 1 mL of cold sterile DPBS. The resulting BALF was used to culture primary alveolar macrophages by incubating the BALF in T-75 tissue-culture flasks in complete culture media (RPMI 1640, 10% fetal bovine serum, 1% penicillin/streptomycin) supplemented with hTGF-β1 (10 ng/mL, Peprotech, #100-21), murine GM-CSF (30 ng/mL, Peprotech #315-03), and rosiglitazone (1 μM, Peprotech, #1227342). Flow cytometry confirmed a phenotypically homogeneous macrophage population and analyses were restricted to cells within established macrophage gates.

### Mouse lung fibroblast (MLF) isolation

Mouse lung fibroblasts (MLFs) were isolated from WT and PAR2 KO C57BL/6J mice obtained from The Jackson Laboratory (8–12 weeks of age). Lungs were lavaged with DPBS and excised, with major airways, vessels, and extraneous tissue carefully removed. Lung tissue was minced and transferred to digestion media (5 mg/mL collagenase [300 U/mg], 25 mg/mL trypsin, 5 mg/mL DNase, DMEM). Digestion was performed two or more times until tissue fragments were adequately dissociated. After digestion, lung fragments were filtered through a 28 mm syringe filter, followed by a 25 mm syringe filter. The isolated MLFs were cultured in T-75 tissue culture flasks with complete media (DMEM supplemented with 10% fetal bovine serum, 1% penicillin/streptomycin). This protocol yields fibroblast-enriched cultures based on adherence, growth characteristics and spindle-shaped morphology. Moreover, we have previously established that these MLFs produce collagen upon TGF-β1 stimulation (Thompson et al., 2015). While minor contamination by other cell types cannot be completely excluded, the functional responses were consistent with a fibroblast phenotype.

### Cell treatment protocol

BMDMs and mexAMs were exposed for 24 h to 10 μg/mL of MWCNTs. Cytotoxicity was assessed by the Pierce LDH Cytotoxicity Assay Kit (ThermoFisher, Waltham, MA). A concentration of 10 μg/mL was selected for *in vitro* experiments based on preliminary dose-response experiments that produced cellular responses without significant cytotoxicity (data not shown). Moreover, this dose of MWCNTs was consistent with other studies that showed little or no significant cytotoxicity to cultured macrophages *in vitro* (Lee et al., 2012; Shipkowski et al., 2015). To simulate a Th2 allergic lung microenvironment, cells were pretreated with 20 ng/mL IL-4 and IL-13 for 24 h before MWCNT exposure. IL-4 and IL-13 concentrations were selected based on prior dose-response optimization, demonstrating induction of M2-associated markers within this timeframe. Cytokine-containing media were replaced with fresh cytokine-free media before MWCNT exposure to ensure that observed effects were attributable to the nanoparticles. To evaluate the effects of MWCNT-treated macrophages on fibroblast activation, MLFs were incubated in macrophage-conditioned media collected after BMDM and mexAM exposures.

### Flow cytometry

Macrophage polarization was assessed by flow cytometry. After 24 h treatments, BMDMs and mexAMs were collected in cold DPBS. Non-specific Fc receptor antibody (Abs) binding was blocked by incubating macrophages in TruStain FcX PLUS (156603; BioLegend) for 10 min. Cells were then washed and stained with a mixture of antimouse fluorochrome-conjugated Abs: Zombie-NIR (423105; BioLegend), CD11b (clone M1/70; BioLegend), CD11c (clone N418; BioLegend), CD206 (clone C068C2; BioLegend), and Arg-1 (clone A1exF5; ThermoFisher). Flow cytometry analysis was performed using a sequential gating strategy to identify macrophage populations. Cells were first gated based on forward scatter height (FSC-H) and side scatter height (SSC-H) to exclude debris and to select cells within the size and granularity range consistent with macrophages. Singlets were then selected by gating SSC-A versus SSC-H. Live cells were identified by exclusion of Zombie-NIR-positive events. Following viability gating, BMDMs were defined as CD11b^+^CD11c^−^ cells and mexAMs were identified as CD11b^−^CD11c^+^ cells. Macrophage polarization markers, Arg-1 and CD206, were analyzed within these gated macrophage populations. Data were collected on a BD LSR II flow cytometer with BD FACSDiva software. Data analysis was performed with FlowJo version 10.0.

### qRT-PCR

RNA was extracted from treated cells, and cDNA was synthesized. The FastStart Universal Probe Master (Rox) (Roche) was used to perform Taqman qPCR on the Applied Biosystems QuantStudio3 Real-Time PCR System Thermal Cycling Block. Expression of *Col1a1*, *Col1a2*, and *Arg-1* was quantified relative to β2-microglobulin (*B2M*) as the endogenous control.

### Immunoblotting

Cell lysates were collected after treatments and lysed in a buffer containing protease and phosphatase inhibitors. Proteins were separated on Mini-PROTEAN TGX 4%–15% SDS-PAGE gels (Bio-Rad Laboratories) and transferred onto PVDF membranes. Membranes were blocked and incubated with mouse primary antibodies purchased from Cell Signaling Technology, including phosphorylated (p)-STAT6 at Tyr640 #56554S; STAT6 #5397S; Arginase-1 #93668S; and β-actin #4967L. Bands were visualized using an Amersham Imager 680 (GE Life Sciences), and semi-quantitative densitometry was performed using ImageJ version 2.0.

### Myeloid-specific PAR2 KO mice

Mice with a conditional PAR2 gene (*Par2* ^*fl/fl*^) were provided as a gift to Dr. Antoniak from Dr. Eric Camerer (INSERM UMR 970, Paris, France). *Par2* ^*fl/fl*^ mice were backcrossed on the C57BL/6J background. Mice were generated as follows. Myeloid cell-specific PAR2-deficient (*Par2* ^*fl/fl*^;Lsym^Cre+^, PAR2^ΔMye^) mice were generated by crossing female *Par2* ^*fl/fl*^ with male *Par2* ^*fl/fl*^ mice expressing lysozyme M (LysM)-dependent Cre recombinase (Gunther et al., 2021). Male PAR2^ΔMye^ mice between 8 and 12 weeks of age were used in this study. Littermate *Par2* ^*fl/fl*^ mice were used as controls. Mice were housed in an AAALAC (Association for Assessment and Accreditation of Laboratory Animal Care) accredited animal facility. All animal procedures were approved by the NC State University Institutional Animal Care and Committee (IACUC).

### Exposure of mice to MWCNTs and HDM extract

Exposure procedures consisted of three sessions in the sensitization phase (days 1, 3, 5) and three sessions in the challenge phase (days 15, 17, 19) and were based on previous studies (Lee et al., 2023; Tisch et al., 2025a; b). Mice were exposed via oropharyngeal aspiration (OPA) to 50 µL of vehicle (PBS), MWCNTs, HDM extract, or a combination of MWCNTs and HDM extract. Each HDM extract dose was 0.5 µg/mouse (0.02 mg/kg), and each MWCNT dose was 12.5 µg/mouse (0.5 mg/kg), resulting in total delivered doses of 0.12 mg/kg HDM extract and 3 mg/kg MWCNTs. A low dose of HDM extract was used in this experiment to demonstrate the synergistic effect of MWCNTs with allergen exposure.

### Necropsy and tissue collection

Necropsy was performed on day 22. Mice were euthanized with an intraperitoneal injection of pentobarbital. BALF was collected from each mouse by cannulating the trachea and conducting lavages of the lungs with 0.5 mL of chilled DPBS two times. BALF was utilized to quantify lung protein, lactate dehydrogenase (LDH), and various cytokines and chemokines. For histopathology, the left lung lobe was fixed in 10% neutral buffered formalin for 24 h, then transferred to 70% ethanol for 1 week before being embedded in paraffin and processed further.

### BALF analyses

The Pierce BCA Protein Assay Kit (ThermoFisher) was used to determine protein concentrations in mouse BALF. As an indicator of pulmonary cytotoxicity, LDH activity in BALF was measured with the Pierce LDH Cytotoxicity Assay Kit (ThermoFisher). Total BALF cell counts were performed using a hemocytometer. For differential cell counts, 100 μL of BALF were centrifuged onto glass slides. Slides were then fixed and stained with the Diff-Quik stain set. Cell differentials were quantified by counting 500 cells per slide using an Olympus light microscope BX41 to determine the relative numbers of cells.

### Quantitative morphometry of airway fibrosis and mucous cell metaplasia

Airway fibrosis was assessed on Masson’s trichrome-stained slides by morphometric analysis using the image processing program developed at the National Institute of Health (NIH), ImageJ version 2.0 (Baidoo et al., 2023; Rueden et al., 2017). This protocol was adapted from Baidoo and colleagues, who used this technique to quantify total collagen content in human colon samples (Baidoo et al., 2023). Total collagen quantification is the proportion of positive pixels or gray values surrounding the airways. All images were acquired at ×100 magnification or approximately a 1500 × 1000 μm (HxW) region using an Olympus BX41 light microscope. Mucous cell metaplasia and airway mucus production were assessed by imaging all airways in each AB-PAS-stained sample. The airway perimeter was manually traced, and AB-PAS+ signal was quantified using the ImageJ Colour Deconvolution 2 plugin, which separates histological dye layers based on AB-PAS color vectors (Lee et al., 2023; Ruifork and Johnston, 2001). Mucin density was then normalized to the total airway area within each field to account for variability in airway size between specimens. Following color deconvolution and normalization, images were thresholded, converted into grayscale, and analyzed for mean pixel density.

### Statistical analysis

One-way ANOVA with Tukey’s *post hoc* test or Student’s t-test was used to assess differences between treatment groups (GraphPad Prism, version 10.0). Two-way ANOVA with Tukey’s *post hoc* test was employed to evaluate differences between genotypes. All data are presented as mean ± SEM of experimental 4–5 replicates or 4–6 mice.

## Results

### MWCNTs exacerbate M2-associated proteins Arg-1 and STAT6 via PAR2 signaling

**FIGURE 2:**
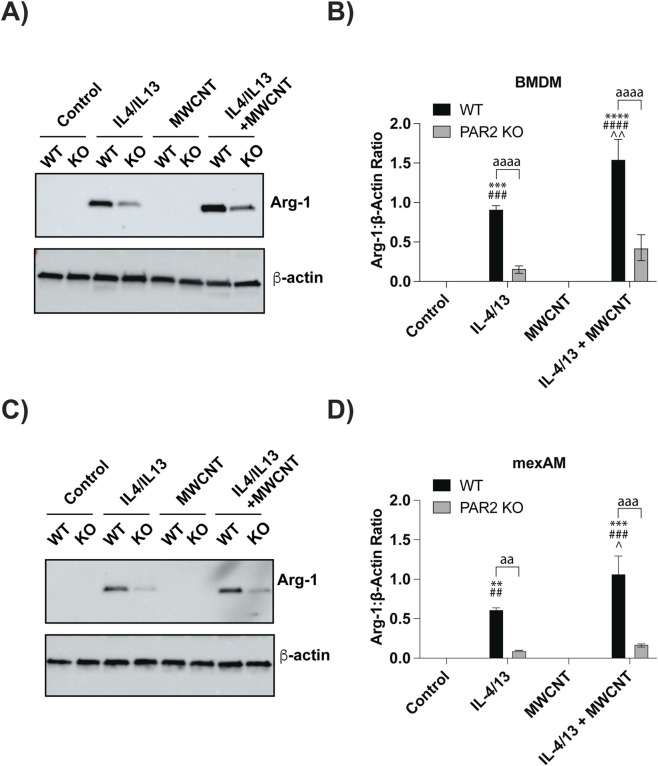
Effects of MWCNTs on Arg-1 protein expression in WT or PAR2 KO BMDMs and mexAMs following 24 h exposure to MWCNTs with or without IL-4/IL-13 pretreatment. **(A)** Representative Western blot showing Arg-1 and β-actin protein expression in BMDMs. **(B)** Densitometric analysis of Arg-1 expression in BMDMs, normalized to β-actin, from three independent experiments. **(C)** Representative Western blot showing Arg-1 and β-actin protein expression in mexAMs under the same treatment conditions. **(D)** Densitometric analysis of Arg-1 expression in mexAMs, normalized to β-actin, from three independent experiments. ^aa^P < 0.01, ^aaa^P < 0.001, ^aaaa^P < 0.0001 between genotypes; ^**^P < 0.01, ^***^P < 0.001, ^****^P < 0.0001 compared to control; ^##^P < 0.01, ^###^P < 0.001, ^####^P < 0.0001 compared to MWCNT; ^P < 0.05, ^^P < 0.01 compared to pretreatment with IL4/IL13. Determined by two-way ANOVA and Tukey’s *post hoc* test. Data are presented as mean ± SEM.

Using the cell isolation protocol shown in (Figure 1A), WT and PAR2 KO BMDMs and mexAMs were pretreated with IL-4 and IL-13 (20 ng/mL each) for 24 h to mimic a Th2 allergic lung microenvironment. After cytokine conditioning, the media were replaced with cytokine-free media, and the cells were then exposed to MWCNTs (10 μg/mL) for an additional 24 h. TEM images of the MWCNTs are shown in Figure 1B. Western blot analysis was performed on cell lysates to evaluate expression of Arg-1 and pSTAT6, two proteins central to M2 polarization and allergic lung disease. As expected, IL-4/IL-13 pretreatment alone increased Arg-1 expression in both WT and PAR2 KO BMDMs and mexAMs (Figures 2A,C). Densitometric analysis revealed that Arg-1 induction was significantly lower in PAR2 KO macrophages compared to WT controls (Figures 2B,D), indicating that PAR2 contributes to Arg-1 regulation. Furthermore, exposure to MWCNTs significantly elevated Arg-1 protein levels in IL-4/IL-13-pretreated WT macrophages (Figures 2B,D), suggesting that MWCNTs exacerbate Arg-1 induction under Th2 allergic conditions. In contrast, this MWCNT-driven increase was blunted in PAR2 KO macrophages, where Arg-1 levels remained comparable to IL-4/IL-13 pretreatment alone (Figures 2B,D). These results indicate that PAR2 mediates the amplification of Arg-1 expression in M2-polarized macrophages following MWCNT exposure.

**FIGURE 3:**
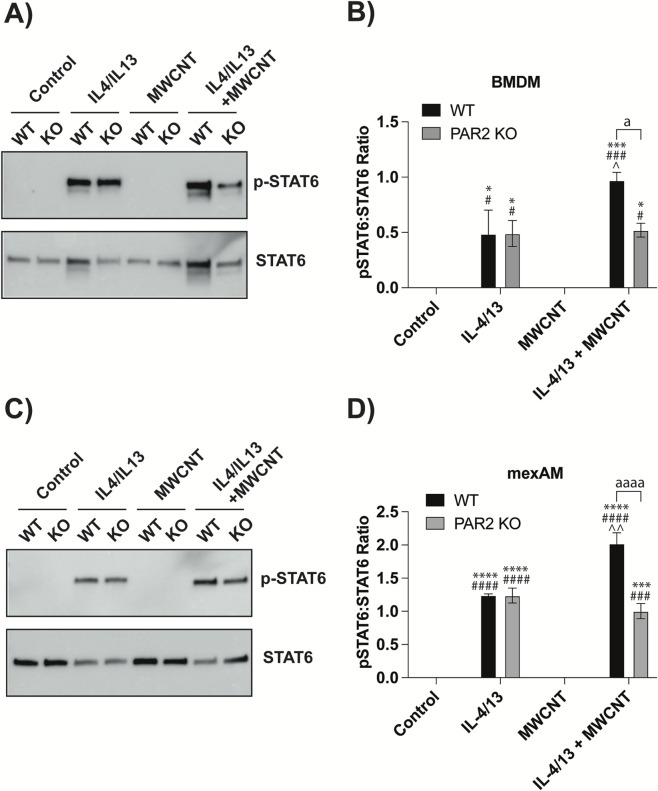
Effects of MWCNTs on pSTAT6 and total STAT6 protein expression in WT and PAR2 KO BMDMs and mexAMs following 24 h exposure to MWCNTs with or without IL-4/IL-13 pretreatment. **(A)** Representative Western blot of pSTAT6 and STAT6 from BMDMs. **(B)** Densitometric quantification of pSTAT6 normalized to total STAT6 in BMDMs, from three independent experiments. **(C)** Representative Western blot of pSTAT6 and STAT6 from mexAMs. **(D)** Densitometric quantification of pSTAT6 normalized to total STAT6 in mexAMs, from three independent experiments. ^a^P < 0.05, ^aaaa^P < 0.0001 between genotypes; *P < 0.05, ***P < 0.001, ****P < 0.0001 compared to control; ^#^P < 0.05, ^###^P < 0.001, ^####^P < 0.0001 compared to MWCNT; ^P < 0.05, ^^P < 0.01 compared to pretreatment with IL4/IL13. Determined by two-way ANOVA and Tukey’s *post hoc* test. Data are presented as mean ± SEM.

Consistent with Arg-1 expression, STAT6, a key driver of Arg-1 transcription, showed a parallel pattern. IL-4/IL-13 pretreatment significantly increased STAT6 phosphorylation in both WT and PAR2 KO BMDMs and mexAMs (Figures 3A,C). Densitometric analysis found no significant differences between WT and PAR2 KO macrophages following IL-4/IL-13 pretreatment alone (Figures 3B,C). However, following IL-4/IL-13 conditioning, MWCNT exposure further increased STAT6 phosphorylation in WT macrophages but not in PAR2 KO macrophages (Figures 3B,D). Collectively, these results demonstrate that MWCNTs enhance Arg-1 expression and pSTAT6 in a PAR2-dependent manner under Th2 allergic conditions.

### PAR2 regulates macrophage polarization through modulation of Arg-1 expression

**FIGURE 4:**
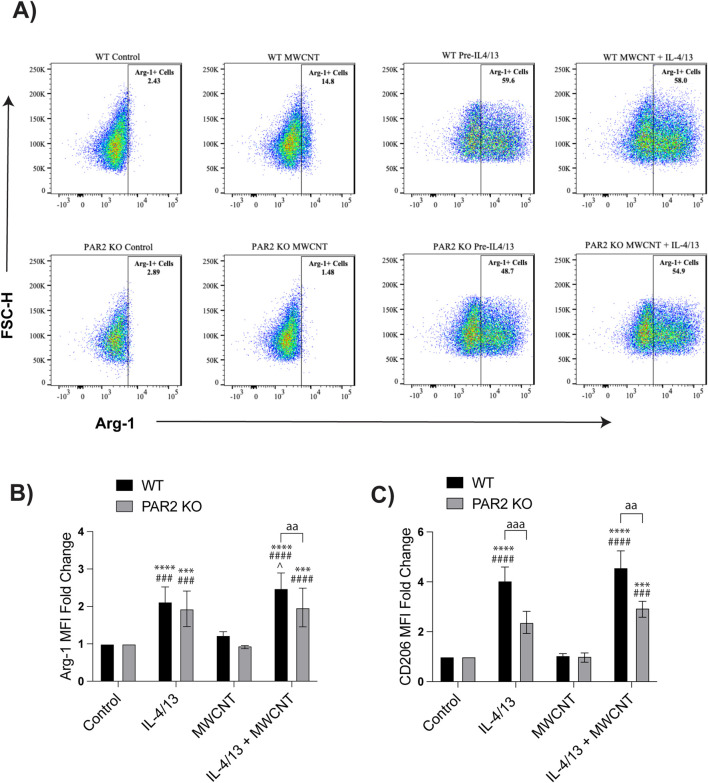
Effects of MWCNTs on Arg-1 and CD206 expression in BMDMs from WT and PAR2 KO mice. Cells were treated with media alone (control), MWCNTs, IL-4/IL-13, or IL-4/IL-13 pretreatment followed by MWCNTs for 24 h. **(A)** Representative flow cytometry scatter plots showing the percentage of Arg-1^+^ BMDMs in each treatment group. **(B)** Mean fluorescence intensity (MFI) of Arg-1 expression in BMDMs, from three independent experiments. **(C)** MFI of CD206 expression in BMDMs, from three independent experiments. ^aa^P < 0.01, ^aaa^P < 0.001 between genotypes; ^***^P < 0.001, ^****^P < 0.0001 compared to control; ^###^P < 0.001, ^####^P < 0.0001 compared to MWCNT; ^P < 0.05 compared to pretreatment with IL4/IL13. Determined by two-way ANOVA and Tukey’s *post hoc* test. Data are presented as mean ± SEM.

Flow cytometry was used to distinguish macrophage subsets and evaluate Arg-1 expression at the single-cell level. BMDMs were identified as CD11b^+^CD11c^−^ cells and mexAMs were identified as CD11b^−^CD11c^+^ cells, while dead cells were excluded using Zombie-NIR. In WT BMDMs, MWCNT exposure increased the overall proportion of Arg-1^+^ cells, whereas this effect was absent in PAR2 KO BMDMs (Figure 4A). Under Th2 conditions, MWCNT exposure minimally altered the percentage of Arg-1^+^ BMDMs compared to IL-4/IL-13 alone, but analysis of Arg-1 mean fluorescence intensity (MFI) revealed a significant increase in WT BMDMs (Figure 4B). This indicates that MWCNTs enhance the per-cell expression of Arg-1 rather than the number of Arg-1^+^ cells. In contrast, PAR2 KO BMDMs showed significantly lower Arg-1 MFI relative to WT across treatments, confirming that PAR2 modulates the degree of Arg-1 expression (Figure 4B). CD206 MFI, another M2 marker, was also increased in WT BMDMs treated with IL-4/IL-14 or IL-4/IL-13 with MWCNTs, while PAR2 KO BMDMs expressed lower CD206 levels (Figure 4C).

**FIGURE 5:**
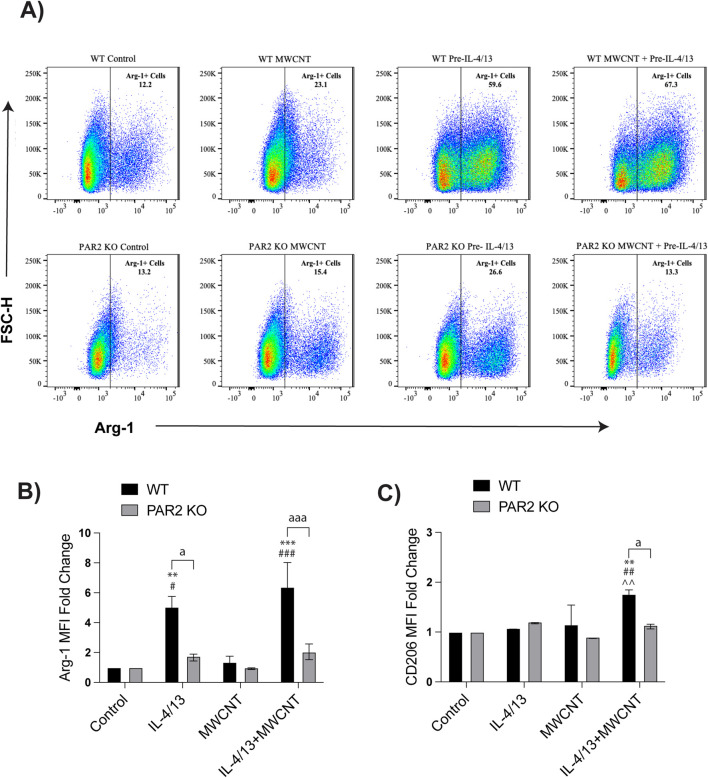
Effects of MWCNTs on Arg-1 and CD206 expression in mexAMs from WT and PAR2 KO mice. Cells were treated with media alone (control), MWCNTs, IL-4/IL-13, or IL-4/IL-13 pretreatment followed by MWCNTs for 24 h. **(A)** Representative flow cytometry scatter plots showing the percentage of Arg-1^+^ mexAMs in each treatment group. **(B)** Mean fluorescence intensity (MFI) of Arg-1 expression in mexAMs, from three independent experiments. **(C)** MFI of CD206 expression in mexAMs, from three independent experiments. ^a^P < 0.05, ^aaa^P < 0.001 between genotypes; ^**^P < 0.01, ^***^P < 0.001 compared to control; ^#^P < 0.05, ^##^P < 0.01, ^###^P < 0.001 compared to MWCNT; ^^P < 0.01 compared to pretreatment with IL4/IL13. Determined by two-way ANOVA and Tukey’s *post hoc* test. Data are presented as mean ± SEM.

In mexAMs, dependence on PAR2 was even more pronounced than in BMDMs. PAR2 KO mexAMs displayed a marked reduction in both the proportion of Arg-1^+^ cells (Figure 5A) and Arg-1 MFI (Figure 5B) compared to WT mexAMs across all conditions. In WT mexAMs, MWCNTs significantly increased Arg-1^+^ cell numbers and Arg-1 MFI following IL-4/IL-13 pretreatment, whereas these responses were diminished in PAR2 mexAMs. CD206 expression was also significantly higher in WT mexAMs treated with IL-4/IL-13 and MCWNTs, while PAR2 KO mexAMs showed little change (Figure 5C). These data indicate that PAR2 plays distinct roles in regulating Arg-1 expression in infiltrating BMDMs versus resident mexAMs, with a stronger reliance on PAR2 observed in the mexAMs.

### PAR2-expressing infiltrating macrophages induce profibrotic gene expression in MLFs

**FIGURE 6:**
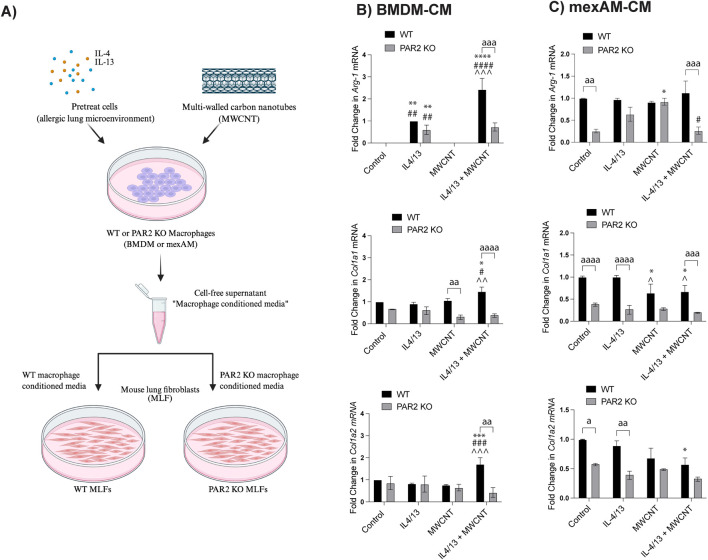
Gene expression in mouse lung fibroblasts (MLF) from WT and PAR2 KO mice following 24 h treatment with conditioned medium from corresponding WT or PAR2 KO BMDMs or mexAM pretreated with IL4/IL13, MWCNTs, or IL4/IL13 pretreatment and MWCNTs. **(A)** Schematic of treatment strategy (Created with Biorender.com). **(B)** *Arg-1*, *Col1a1*, and *Col1a2* mRNA expression in MLFs treated with BMDM conditioned media. **(C)** *Arg-1*, *Col1a1*, and *Col1a2* expression in MLFs treated with mexAMs conditioned media. ^a^P < 0.05, ^aa^P < 0.01, ^aaa^P < 0.001, ^aaaa^P < 0.0001 between genotypes; ^*^P < 0.05, ^**^P < 0.01, ^***^P < 0.001 compared to control; ^#^P < 0.05, ^##^P < 0.01, ^###^P < 0.001, ^####^P < 0.0001 compared to MWCNT; ^P < 0.05, ^^P < 0.01, ^^^P < 0.001 compared to pretreatment with IL4/IL13. Determined by two-way ANOVA and Tukey’s *post hoc* test. Data are presented as mean ± SEM.

**FIGURE 7:**
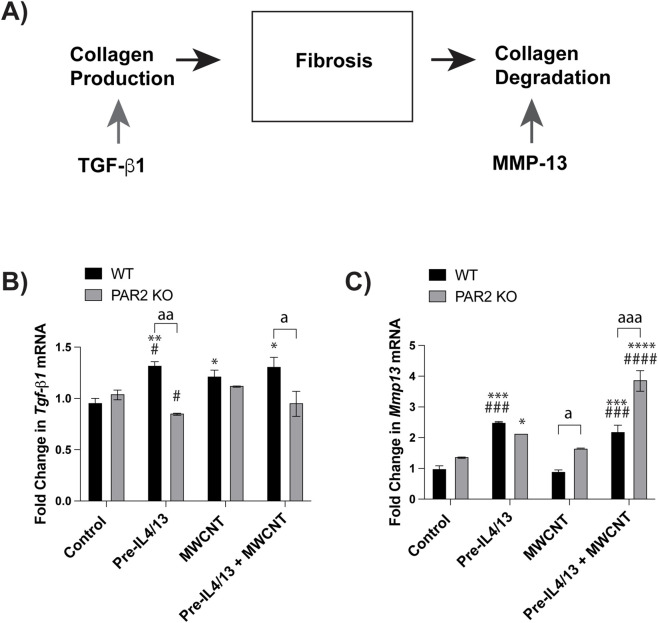
Gene expression in BMDMs pretreated with IL4/IL13, MWCNTs, or IL4/IL13 pretreatment and MWCNTs. **(A)** WT BMDMs promote collagen production while PAR2 KO BMDMs encourage collagen degradation. **(B)** *Tgf-β1* mRNA expression in BMDMs. **(C)** *Mmp13* mRNA expression in BMDMs.

To determine whether MWCNT-exposed macrophages promote fibroblast activation, MLFs were treated with conditioned media from BMDMs or mexAMs previously exposed to MWCNTs and IL-4/IL-13 (Figure 6A). We then examined the expression of genes associated with a profibrotic fibroblast phenotype. qRT-PCR analysis revealed that conditioned media from WT BMDMs exposed to IL-4/IL-13 and MWCNTs induced significant upregulation of the expression of *Arg-1*, *Col1a1*, and *Col1a2* in MLFs compared to media from untreated BMDMs (Figure 6B). In contrast, conditioned media from PAR2 KO BMDMs failed to induce significant increases in *Arg-1*, *Col1a1*, and *Col1a2*, demonstrating that PAR2 expression in macrophages is required for fibroblast activation (Figure 6B). Conversely, MLFs treated with conditioned media from mexAMs showed little to no change in *Arg-1*, *Col1a1*, and *Col1a2* expression across treatment groups (Figure 6C), highlighting a limited role for alveolar macrophages in fibroblast activation. Notably, fibroblast responsiveness was also influenced by PAR2 status, as MLFs derived from PAR2 KO mice exhibited attenuated gene expression compared with WT MLFs (Figure 6). Together, these findings suggest that infiltrating macrophages represented by BMDMs, but not resident alveolar macrophages represented by mexAMs, drive PAR2-dependent fibroblast activation and profibrotic signaling following MWCNT exposure. As illustrated in Figure 7A, fibrosis is determined by the balance between collagen production stimulated by factors such as TGF-β1 and the degradation of collagen mediated by factors such as MMP13. The expression of mRNA encoding the profibrotic gene *Tgf-β1* was significantly increased in WT BMDMs compared to PAR2 KO BMDMs (Figure 7B). Additionally, PAR2 KO BMDMs significantly upregulate the collagen-degrading enzyme *Mmp13* compared to the WT BMDMs (Figure 7C).

### Allergen and MWCNT-induced lung remodeling in mice requires PAR2 signaling in myeloid cells

**FIGURE 8:**
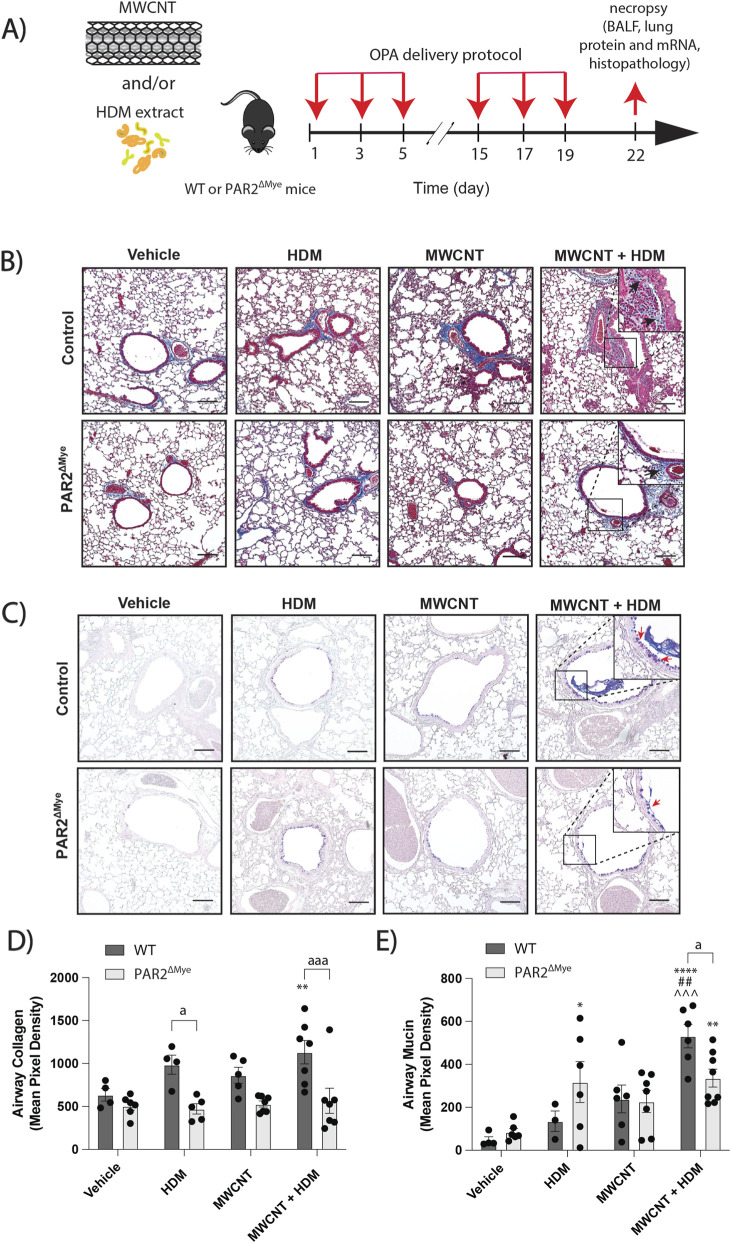
The effects of myeloid-specific PAR2 deficiency (PAR2^ΔMye^) on allergic lung disease in mice exposed to MWCNTs, HDM extract, or both. **(A)** Illustration of the exposure protocol. **(B)** Representative images of Masson’s trichrome-stained lung sections showing blue-stained collagen. Black bars = 100 μm. Insert panels show higher magnification of blue stained, trichrome-positive airway collagen (black arrows). **(C)** Representative images of AB-PAS-stained lung tissue sections positive for PAS+ mucins indicated by purple staining. Black bars = 100 μm. Insert panels show higher magnification of purple-stained, AB-PAS-positive goblet cells (red arrows). **(D)** Quantification of trichrome-positive airway collagen. **(E)** Quantification of AB-PAS+ airway mucin. Black bars = 100 μm. ^a^P < 0.05, ^aaa^P < 0.001 between genotypes; ^*^P < 0.05, ^**^P < 0.01, ^****^P < 0.0001 compared to control; ^##^P < 0.01 compared to MWCNT; ^^^P < 0.001 compared to pretreatment with IL4/IL13. Determined by two-way ANOVA and Tukey’s *post hoc* test. Data are presented as mean ± SEM; each data point is an individual mouse.

To validate the *ex vivo* findings *in vivo* and to determine whether macrophage PAR2 signaling contributes to lung pathology, we used a myeloid-specific PAR2 knockout (PAR2^ΔMye^) mouse model. WT and PAR2^ΔMye^ mice were exposed via OPA to HDM extract, MWCNTs, or a combination of both during a sensitization phase (days 1, 3, 5) and a challenge phase (days 15, 17, 19) (Figure 8A). Lungs were harvested after the final exposure for histopathological evaluation.

Airway fibrosis was measured by quantitative morphometric analysis on Masson’s trichrome-stained lung sections. No significant differences in airway collagen deposition were seen in mice exposed to HDM extract or MWCNTs alone. However, WT mice co-exposed to MWCNTs and HDM extract displayed significantly greater airway collagen compared with vehicle control cohorts (Figure 8B). Interestingly, this effect was reduced in PAR2^ΔMye^ mice, which showed significantly less airway collagen deposition in the co-exposure group compared to WT mice (Figures 8B,D). Additionally, AB-PAS staining was performed to evaluate airway mucus production. WT mice co-exposed to MWCNTs and HDM extract showed a notable increase in AB-PAS-positive mucus compared to vehicle-treated controls (Figures 8C,E). Although PAR2^ΔMye^ mice also showed elevated mucus levels following co-exposure, the level of mucus production was significantly lower than in WT mice (Figures 8C,E). Additionally, BALF analysis demonstrated that co-exposure significantly increased total protein, LDH, eosinophilia, and total cell numbers in WT and PAR2^ΔMye^ with no significant differences (Supplementary Image 2). While these measures of allergic lung disease increased regardless of genotype, airway fibrosis and mucus hypersecretion were selectively reduced in PAR2^ΔMye^ mice. These data demonstrate that PAR2 expression in myeloid cells, such as macrophages, is essential for promoting airway remodeling, highlighting PAR2 as a central driver in allergic lung disease pathology.

## Discussion

This study aimed to evaluate the mechanisms by which MWCNTs influence macrophage polarization during allergic lung disease and to determine whether these macrophages contribute to a profibrotic lung environment. Additionally, we investigated the role of PAR2 in these processes using both *ex vivo* PAR2-deficient macrophages and myeloid-specific PAR2 knockout mice *in vivo*. Given that allergic lung disease is characterized by elevated type 2 cytokines such as IL-4 and IL-13, we pretreated macrophages with recombinant murine IL-4/IL-13 to simulate a Th2-skewed microenvironment. In this *in vitro* system, we found that exposure to MWCNTs amplifies Th2-driven M2 macrophage polarization in a PAR2-dependent manner, leading to enhanced fibroblast activation and airway remodeling. These findings identify macrophages as key cellular mediators linking MWCNT exposure to fibrotic outcomes in allergic lung disease.

MWCNTs are considered toxicologically significant inhaled particles due to their high aspect ratio, size, and biopersistence in the lung. Following inhalation, MWCNTs deposit in the distal airways and alveolar regions, where they are inefficiently cleared and can remain for prolonged periods (Duke and Bonner, 2018). This persistence raises concern for chronic biological effects rather than acute inflammatory responses. As a result, MWCNTs are recognized as occupational hazards, and the National Institute for Occupational Safety and Health (NIOSH) has established recommended exposure limits to reduce long-term respiratory risk (NIOSH, 2013). Importantly, increasing evidence in experimental animal models indicates that these materials can exacerbate allergic lung diseases and disrupt normal immune and tissue repair processes (Lee et al., 2023; Tisch et al., 2025a; Tisch et al., 2025b). Consequently, MWCNT exposure poses a significant health risk to susceptible populations, including individuals with asthma.

Within this toxicological context, macrophages are central determinants of MWCNT-induced lung pathology, as they represent primary cells responsible for particle recognition and activation of downstream immune and tissue responses. Because macrophage-mediated signaling is a key driver of airway remodeling during allergic lung disease, we next extended these findings *in vivo* using a myeloid-specific PAR2 knockout exposed to HDM extract, a clinically relevant allergen, and MWCNTs to mimic co-exposure scenarios observed in environmental and occupational settings. Of note, commercially available HDM extracts contain measurable levels of endotoxin, which is known to contribute to innate immune activation. However, the administered dose was intentionally low to reveal synergistic effects with MWCNTs. Our group has previously shown that combined exposure to HDM extract and MWCNTs exacerbates hallmark features of allergic lung disease, including eosinophilic inflammation, airway fibrosis, and mucous cell metaplasia compared to either treatment alone (Lee et al., 2023; Tisch et al., 2025a; Tisch et al., 2025b). In the current study, we demonstrate that deletion of PAR2 in myeloid cells significantly reduces airway fibrosis and mucous cell metaplasia, supporting a central role for PAR2-expressing macrophages in mediating airway remodeling during allergic lung disease. These results are consistent with studies showing that PAR2 signaling amplifies airway inflammation and remodeling in response to allergens and environmental triggers (Davidson et al., 2013; Asaduzzaman et al., 2015; Steven et al., 2013).

Our *in vitro* findings demonstrate that MWCNTs significantly enhance M2-like polarization in both BMDMs and mexAMs under Th2 conditions, as evidenced by amplified Arg-1 expression and STAT6 phosphorylation. These effects were attenuated in PAR2 KO macrophages, indicating a key role of PAR2 in promoting or sustaining alternative macrophage activation. Importantly, M2 macrophages have been implicated in driving chronic lung diseases, including pulmonary fibrosis, due to their profibrotic and immunomodulatory functions (Bahram et al., 2025; Kishore and Petrek, 2021). Supporting this, a recent study has shown that reducing M2 macrophage infiltration into the lungs of mice can protect against bleomycin-induced pulmonary fibrosis (Pan et al., 2021). While MWCNTs have previously yielded mixed results with respect to macrophage polarization, such variability likely reflects differences in nanoparticle properties and the immunologic context of exposure (Dong and Ma, 2018b; Lim et al., 2020; Allegri et al., 2016). In our model, MWCNTs enhanced IL-4/IL-13-driven M2 macrophage polarization, suggesting that MWCNTs may exacerbate the severity of allergic lung disease by amplifying Th2-associated macrophage activation. One possible mechanism involves crosstalk between PAR2 and Toll-like receptor 4 (TLR4). Prior studies have shown that PAR2 interactions with TLR4 can inhibit or enhance downstream signaling depending on the experimental conditions (Widera et al., 2019; Nhu et al., 2012). For example, the conjoint activation of PAR2 and TLR4 signaling resulted in M2 macrophage polarization and the inhibition of proinflammatory molecules, such as TNFα and IL-6 (Nhu et al., 2012). Our findings are consistent with this model, suggesting PAR2 sustains STAT6 signaling and Arg-1 expression in M2-skewed macrophages, whereas in the absence of PAR2, unopposed TLR4 activation may favor incomplete or proinflammatory macrophage activation. Beyond TLR4, PAR2 is also known to cooperate with PAR1 in fibroblast signaling (Lin et al., 2015), and to modulate NF-κB-dependent inflammatory pathways (Shpacovitch et al., 2007), further underscoring its role as a regulator of macrophage plasticity.

Given that M2 macrophages secrete mediators that regulate tissue remodeling, we next investigated whether PAR2-dependent macrophage polarization contributes to fibroblast activation. Using macrophage-conditioned media, we found that PAR2-expressing BMDMs, but not mexAMs, significantly induced expression of profibrotic genes in MLFs, including *Arg-1, Col1a1*, and *Col1a2*. These results support the prior research demonstrating that infiltrating monocyte-derived macrophages are the principal contributors to fibroblast activation during lung injury, whereas resident alveolar macrophages play a more homeostatic role (Byrne et al., 2016; Misharin et al., 2013). For example, the specific depletion of monocyte-derived macrophages following lung injury has been shown to reduce the severity of pulmonary fibrosis (Misharin et al., 2017), highlighting the importance of this macrophage subset in fibrotic lung diseases. In addition to the role of PAR2 in macrophages, we also observed that PAR2 deficiency in lung fibroblasts markedly attenuated their response to profibrotic cues. MLFs derived from PAR2 KO mice exhibited significantly lower expression of *Arg-1*, *Col1a1*, and *Col1a2* following stimulation with BMDM-conditioned media, suggesting that PAR2 in fibroblasts may also regulate key signaling pathways involved in fibroblast activation and collagen deposition. Prior studies have demonstrated that PAR2 can transactivate TGF-β receptor signaling, which is known to mediate fibroblast activation and extracellular matrix production leading to fibrosis (Chung et al., 2013). Together, these findings highlight a critical role of PAR2 in mediating macrophage-driven fibroblast activation, showing that PAR2 not only regulates polarization of macrophages but also directly influences fibroblast responsiveness to macrophage-derived signals and collagen synthesis.

M2 macrophages are widely recognized as key regulators of fibrotic remodeling in the lung, where polarization is driven by Th2 cytokines such as IL-4 and IL-13 through the PI3K-Akt and JAK1-STAT6 signaling pathways (Van Dyken and Locksely, 2013; Will et al., 2008). Our findings demonstrate that MWCNTs enhance M2 polarization in macrophages exposed to IL-4/IL-13, an effect that is significantly diminished in PAR2-deficient macrophages. These results suggest that PAR2 may sustain or amplify STAT6 or PI3K-Akt activation during Th2-induced macrophage polarization. These signaling axes converge on the regulation of a hallmark metabolic enzyme of M2 macrophages, Arg-1. Arg-1 defines the M2 phenotype as well as contributes metabolically to fibrosis by converting arginine to ornithine, a precursor for proline and hydroxyproline, key amino acids in collagen biosynthesis (Van Dyken and Locksley, 2013; Wills-Karp and Finkelman, 2008; Minutti et al., 2017). These connections help link macrophage polarization to collagen biosynthesis. However, while our data strongly support the functional importance of PAR2 and macrophages in lung pathology, it is essential to acknowledge that the myeloid lineage encompasses other cell types beyond macrophages, such as monocytes and neutrophils (Kawamoto and Minato, 2004), which also contribute to allergic inflammation and fibrotic remodeling. Therefore, the myeloid-specific PAR2 knockout model may not directly mirror the effects observed in isolated macrophage populations *ex vivo*. Future studies using cell-type-specific PAR2 deletions will help define the relative contributions of these cell subsets.

These findings highlight PAR2 as a central receptor linking allergen proteases, engineered nanomaterials, and airway remodeling. When in the lungs, MWCNTs disrupt normal immune and repair processes by persisting within lung tissue and altering macrophage to fibroblast communication and promoting pathological remodeling. These effects may manifest as sustained fibroblast activation and collagen deposition (He et al., 2011; Duke and Bonner, 2018; Murphy et al., 2012). Combined with allergen proteases, this creates a setting where PAR2 signaling may promote or sustain chronic remodeling. Looking forward, there are important aspects of this research that need to be addressed. Additional mechanistic work is needed to define how PAR2 interacts with other signaling pathways, including TLR4 and PAR1, to regulate macrophage plasticity and fibroblast activation in the context of allergen and nanoparticle co-exposure. Additionally, the translational relevance of these findings could be advanced by extending analyses to human macrophages and fibroblasts, where PAR2 signaling may similarly amplify fibrotic pathways. Finally, because pharmacological inhibition of PAR2 has already been shown to attenuate allergic airway disease (Asaduzzaman et al., 2015; Heuberger and Schuepbach, 2019; Tisch et al., 2025b), testing PAR2 antagonists in this setting of environmental and occupational MWCNT exposure may provide a path toward therapeutic intervention.

## Conclusion

In conclusion, this study identifies PAR2 as a key regulator of macrophage polarization and profibrotic signaling in the lung during allergic lung disease. We demonstrate that MWCNTs enhance Th2-driven M2 polarization in a PAR2-dependent manner, resulting in increased fibroblast activation and tissue remodeling. Our findings suggest that PAR2 signaling in myeloid cells, especially macrophages, plays a crucial role in mediating the fibrotic response to environmental particulate and allergenic exposures. These results also extend previous observations that PAR2-deficient mice are protected from fibrotic remodeling (Lee et al., 2023; Tisch et al., 2025a) and highlight PAR2 as a central receptor linking nanoparticle exposure, allergen proteases, and immune cell reprogramming. Therefore, these findings not only demonstrate a critical role for PAR2 in macrophage-driven fibrosis but also open avenues for targeted therapies aimed at mitigating environmentally exacerbated allergic lung disease.

## Data Availability

The original contributions presented in the study are included in the article/Supplementary Material, further inquiries can be directed to the corresponding author.
