# Supplementary figures and images for: Role of the protease-activated receptor 2 in multi-walled carbon nanotube-induced macrophage polarization ex vivo and airway fibrosis in murine allergic lung disease in vivo
Source: Front Toxicol. 2026 Feb 18;8:1751684. doi: 10.3389/ftox.2026.1751684 (PMC12957074; doi:10.3389/ftox.2026.1751684)

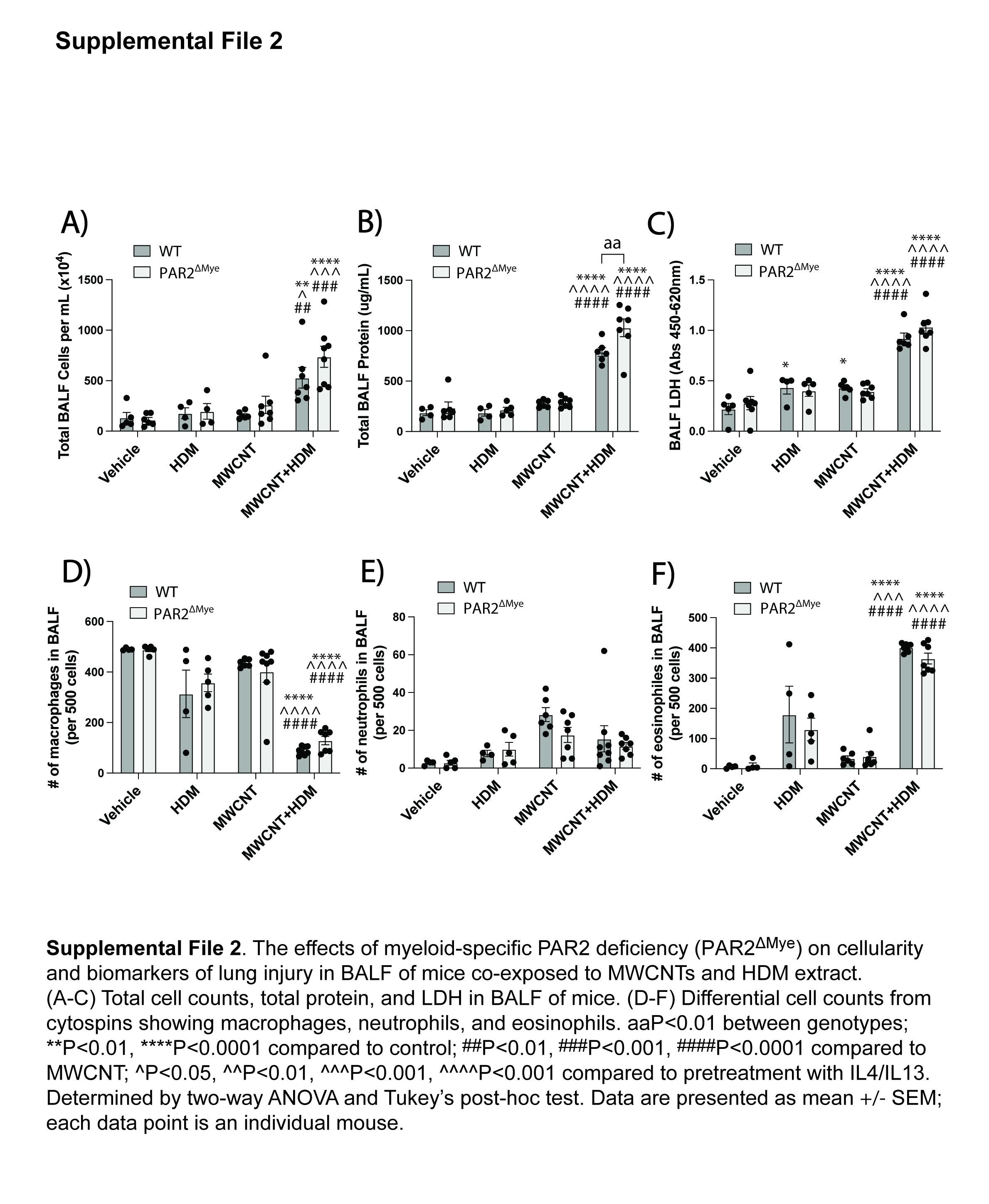

Supplement: Supplementary file 2 [file Image2.tif]
